# Combined Vascular and Orthopaedic Approach for a Pseudotumor Causing Deep Vein Thrombosis after Metal-on-Metal Hip Resurfacing Arthroplasty

**DOI:** 10.1155/2015/926263

**Published:** 2015-09-20

**Authors:** Hossam Abdel-Hamid, Jonathan Miles, Richard W. J. Carrington, Alister Hart, Alex Loh, John A. Skinner

**Affiliations:** Joint Reconstruction Unit (JRU), Royal National Orthopaedic Hospital, Stanmore, Middlesex HA7 4LP, UK

## Abstract

*Introduction*. Metal-on-metal (MoM) hip resurfacings have been associated with a variety of complications resulting from adverse reaction to metal debris. Pseudotumors have rarely been reported to cause deep venous thrombosis (DVT). *Study Design*. A case report and a review of the literature. *Case Presentation*. A 75-year-old female who had left metal-on-metal hip resurfacing 6 years ago presented with left groin pain associated with unilateral lower limb edema and swelling. By duplex and MRI studies, our patient had an extensive soft tissue necrosis associated with a large pelvic mass causing extensive DVT of the lower limb secondary to mechanical compression of the left iliac vein. *Results*. Our case was initially treated for DVT followed by dual surgical approach. The pseudotumor was excised through a separate iliofemoral approach and revision of the hip implant was undertaken through a posterior approach in the same setting. An inferior vena cava (IVC) filter was inserted to minimise the perioperative risks of handling the iliac veins. *Conclusion*. A combined approach with vascular surgeons is required. Combined resection of the pseudotumor and revision of the metal bearing surfaces is essential, in order to achieve a good surgical outcome in this rare complication.

## 1. Introduction

Metal-on-metal (MoM) hip resurfacing was popularised in the late 1990s. Pseudotumors or soft tissue reactions, so-called adverse local tissue reactions (ALTRs), have been reported as complication. These effects are thought to be due to local tissue necrosis or possibly hypersensitivity reactions.

We present a case of large pelvic pseudotumor associated with MoM hip resurfacing resulting in deep vein thrombosis (DVT). Such cases have been described in association with polyethylene debris but rarely reported with resurfacing hip arthroplasty.

## 2. Case Presentation

A 75-year-old female presented to our hospital in April 2014 with left groin pain associated with unilateral lower limb edema and swelling for four weeks.

She had previously had left hip resurfacing arthroplasty in 2008, using Cormet prosthesis (Corin, Cirencester, United Kingdom). She had no history of febrile illness. Metal ion levels were within the normal range recommended by MHRA (Cobalt 2.16 ppb, chromium 2.58 ppb). A metal artifact reduction sequence (MARS) MRI scan of both hips was also performed one year ago. This showed a cystic lesion measuring 30 mm × 25 mm in size. Our patient was asymptomatic with regard to both hips and mobilizing independently and was fully active prior to the onset of recent symptoms.

On examination, the left leg was swollen from thigh to ankle. She had generalized tenderness starting from the groin and the lateral aspect of the thigh radiating down her leg. Her hip arthroplasty wound was well healed, and there were no inflammatory changes. Her hip was painful on all movements, but worse with rotation in flexion. Patient also showed signs of psoas irritation.

On abdominal examination, there was fullness in the left iliac fossa with tenderness on deep palpation. Her right hip examination was normal.

Investigations revealed an elevated erythrocyte sedimentation rate (ESR) of 48 mm/hour and C-reactive protein (CRP) of 37 mg/L (normal: 0–5 mg/L). Her white blood cell count was 5.35 (3.5–11). Metal ion levels were within the normal range, cobalt 1.37 ppb and chromium 2.05 ppb.

**Figure 1:**
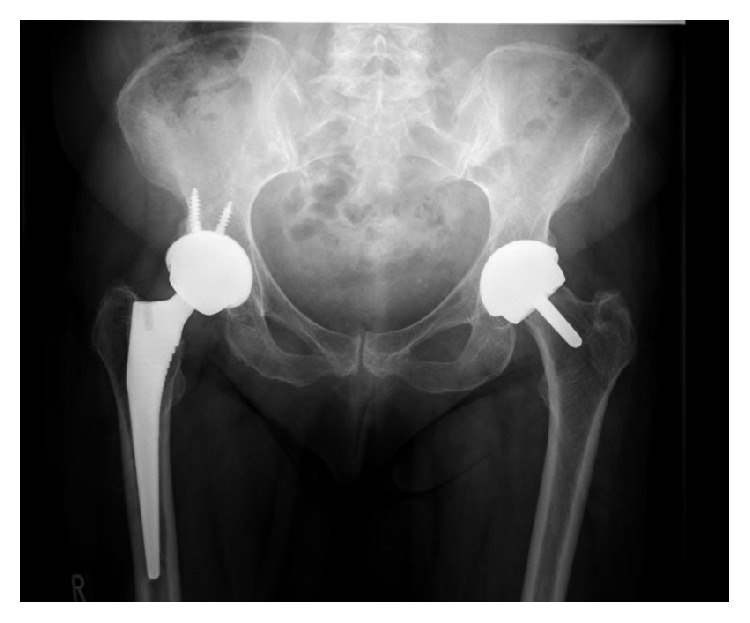
Anteroposterior X-ray pelvis showing right THR and left hip resurfacing.

She was admitted to hospital. A plain radiograph of her pelvis showed satisfactory positioning of right uncemented total hip replacement with 36 mm metal-on-metal articulation (Corail/Pinnacle) and left hip resurfacing, without adverse features (Figure 1).

Duplex ultrasound of the left leg venous system showed a distended occluded left common femoral vein which failed to respond to respiratory excursions. There was extensive induration and oedema of the skin overlying the thigh which hampers good visualisation of the deep veins within the thigh. Also, ultrasound showed an 80 mm × 32 mm left sided psoas collection which extends from just about the level of the femoral head to the level of the lesser trochanter distally. This collection was compressing the left iliac vein.

**Figure 2:**
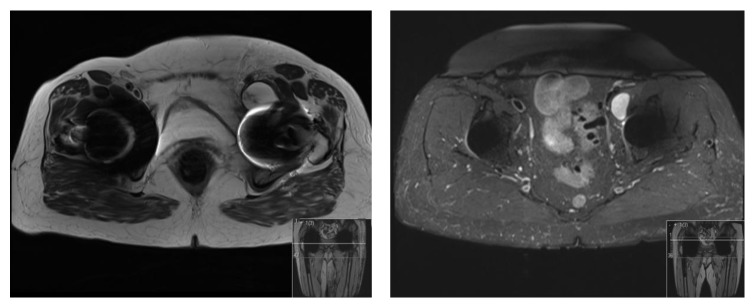
MARS MRI showing a left retroperitoneal cystic mass lesion (74 mm × 32 mm) noted anteriorly and extending into the iliopsoas bursa.

MARS MRI scan of both hips and pelvis showed left retroperitoneal pelvic cystic lesion (74 mm × 32 mm) previously noted anteriorly and now extending into the iliopsoas bursa. It was thin walled and contained low signal linear debris. The external iliac vessels were displaced and flattened. Further, a smaller cystic lesion was also noted around the greater trochanter (20 mm in length × 5 mm in width) (Figure 2).

Our patient was anticoagulated with low molecular weight heparin (LMWH) and warfarin. The swelling improved rapidly with treatment and rest. Revision surgery was planned but delayed for 3 months pending treatment of DVT.

**Figure 3:**
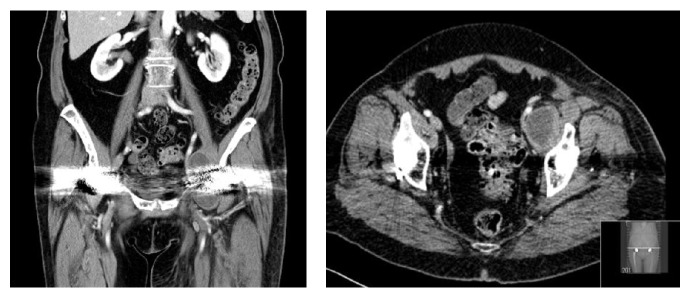
CT angiography showing a large cystic mass compressing the left iliac vessels.

Prior to surgery, a temporary IVC filter was inserted to minimise the risks of intraoperative pulmonary emboli and CT angiography performed before surgery (Figure 3).

At surgery, pseudotumor was resected after mobilisation of the iliac veins, via an extraperitoneal iliofemoral approach by a vascular surgeon. The wound was then closed and the patient placed in the right lateral decubitus position for revision of the left hip resurfacing to total hip replacement via a posterior approach.

Intraoperatively, the external iliac vessels lie closely related to the external wall/capsule of the pseudotumor. The periarticular tissues at the left hip were abnormal and dull grey in colour. Samples were sent for histopathology and microbiology examination. Cultures were sterile after 14 days of incubation. Histologically, the tissue consisted of fibrous and fibrohyalinized tissue with evidence of acute inflammation, predominantly macrophages with only occasional lymphocytes. There were small areas of synovial hyperplasia and focal necrosis. This suggested an adverse reaction to metal debris.

Subsequently, she also completed her course of anticoagulants, and a repeat sonogram revealed patent deep venous system. Her symptoms have significantly improved. She is able to carry out most of her daily activities and has an Oxford score of 35 out of 48 nine months after surgery.

## 3. Discussion

Metal-on-metal hip resurfacing was introduced in the 1990s with the hope that wear rates would be lower for the harder bearing surfaces, that larger head sizes would reduce the rate of dislocation, and that subsequent revision surgery would benefit from bone preservation of the femoral neck [1].

Although there is less wear in MoM bearing compared with metal-on-polyethylene bearings, the particle size in MoM hips is small and may induce greater biological reactivity [2].

Excellent results have been reported after MoM hip resurfacing, but concern regarding abnormal reaction to metal debris in some patients persists. Pandit et al. first described neither malignant nor infective cystic and solid masses associated with metal-on-metal resurfacing and introduced the term pseudotumor. They reported that pseudotumor may be the result of a toxic effect on cells due to particulate wear debris or an idiosyncratic response to the release of metal particles [3].

By 7-year follow-up, the incidence of pseudotumors after hip resurfacing arthroplasty has been described at 0.3% to 3.4% [4–6]. According to Pandit et al., the overall incidence of pseudotumors in MoM resurfacing arthroplasty is 1% at 5 years [3].

Factors associated with higher prevalence of pseudotumors are female sex, bilateral resurfacing arthroplasties, excessive cup anteversion, cup inclination of more than 50°, and higher metal ion levels and some prosthesis types [5, 6].

Pseudotumors may be asymptomatic. Various complications associated with pseudotumors include persistent pain, femoral or sciatic nerve palsy, rash, and ureteral obstruction [7, 8]. Although the DVT from a pseudotumor caused by metal-on-polyethylene hip arthroplasty has been described in the literature [9, 10], only very few cases were described in relation to the large MoM resurfacing arthroplasty.

Our patient had bilateral MoM bearing hips. She remained asymptomatic for 6 years. She developed a pseudotumor that caused local compression of the iliac veins. This is a rare cause of DVT.

Only four cases of such tumors causing DVT after MoM have been described before. One case had a revision surgery and excision of the pseudotumor using the same approach [11]. Another case was deemed to be inoperable and only revision of the implant was done [12]. The third case had only marginal resection of the cystic lesion but ten months later the patient developed recurrent swelling and underwent revision surgery of the implant [13]. The fourth case underwent resection of the pseudotumor only [14].

Our approach to this case was initial treatment of the DVT followed by interval resection of the pseudotumor with IVC filter insertion to minimise the perioperative risks of handling the iliac veins. At the same operation, the hip was revised to a non-metal bearing (ceramic-on-polyethylene) total hip replacement, which removes the metal ion/particle generating force. Nine months after surgery, the patient reported complete relief of groin pain and good function and had an Oxford score of 35 out of 48.

This case report is the first, to our knowledge, to describe a dual surgical approach for management of pseudotumor causing deep venous thrombosis due to mass effect. The external iliac vessels and femoral neurovascular bundles were at risk and lie closely related to the external wall/capsule of the pseudotumor. This study highlights the importance of two surgical specialties working together to reduce the risk of harm to these structures.
